# Anticancer Potential of Sulfonamide Moieties via In-Vitro and In-Silico Approaches: Comparative Investigations for Future Drug Development

**DOI:** 10.3390/ijms24097953

**Published:** 2023-04-27

**Authors:** Tanveer A. Wani, Seema Zargar, Hamad M. Alkahtani, Nojood Altwaijry, Lamees S. Al-Rasheed

**Affiliations:** 1 Department of Pharmaceutical Chemistry, College of Pharmacy, King Saud University, P.O. Box 2457, Riyadh 11451, Saudi Arabia; 2 Department of Biochemistry, College of Science, King Saud University, P.O. Box 22452, Riyadh 11451, Saudi Arabia

**Keywords:** anticancer activity, hearing sperm DNA, computational methods, molecular docking, DNA-binding

## Abstract

Several kinds of anticancer drugs are presently commercially accessible, but low efficacy, solubility, and toxicity have reduced the overall therapeutic indices. Thus, the search for promising anticancer drugs continues. The interactions of numerous essential anticancer drugs with DNA are crucial to their biological functions. Here, the anticancer effects of N-ethyl toluene-4-sulphonamide (**8a**) and 2,5-Dichlorothiophene-3-sulphonamide (**8b**) on cell lines from breast and cervical cancer were investigated. The study also compared how these substances interacted with the hearing sperm DNA. The most promising anticancer drug was identified as 2,5-Dichlorothiophene-3-sulfonamide (**8b**), which showed GI_50_ of 7.2 ± 1.12 µM, 4.62 ± 0.13 µM and 7.13 ± 0.13 µM against HeLa, MDA-MB231 and MCF-7 cells, respectively. Moreover, it also exhibited significant electrostatic and non-electrostatic contributions to the binding free energy. The work utilized computational techniques, such as molecular docking and molecular dynamic (MD) simulations, to demonstrate the strong cytotoxicity of 2,5-Dichlorothiophene-3-sulfamide (**8b**) in comparison to standard Doxorubicin and cisplatin, respectively. Molecular docking experiments provided additional support for a role for the minor groove in the binding of the 2,5-Dichlorothiophene-3-sulfamide (**8b**)-DNA complex. The molecular docking studies and MD simulation showed that both compounds revealed comparable inhibitory potential against standard Doxorubicin and cisplatin. This study has the potential to lead to the discovery of new bioactive compounds for use in cancer treatment, including metallic and non-metallic derivatives of 2,5-Dichlorothiophene-3-sulfonamide (**8b**). It also emphasizes the worth of computational approaches in the development of new drugs and lays the groundwork for future research.

## 1. Introduction

One-in-four fatalities in the United States is caused by cancer, making it the second-leading cause of mortality in the country. Because of its high mortality rate, cancer has been the subject of intensive research to find effective anticancer drugs. Current improvements in oncology have resulted in improved cancer treatment outcomes and increased survival rates [1]. Yet issues like drug resistance, toxicity, and ineffectiveness continue to drive the demand for innovative anticancer therapy [2]. Despite initial success in treating localized cancer, the disease often spreads to other parts of the body [3]. In developing countries, breast cancer is the leading cause of death. New tumors, unusual bleeding, a persistent cough, unexplained weight loss, and changes in bowel habits are all potential cancer signs and symptoms [4,5]. Available anticancer therapies may not always be successful due to drug resistance, low solubility, toxicity, and poor absorption. To beat back these challenges and improve cancer patients’ prognoses, we need better and more effective anticancer drugs. Among the methods under investigation include immunotherapy, gene therapy, and medicines based on nanotechnology. These strategies aim to decrease toxicity, boost therapeutic efficacy, and overcome resistance by more precisely targeting cancer cells [6,7]. The exact molecular interactions of some DNA-acting anticancer medicines with DNA have been studied using structural methods such as high-resolution X-ray diffraction and nuclear magnetic resonance spectroscopy throughout the past decade [8]. Important details about DNA conformation and interactions between drugs and DNA have been revealed by these findings. Particularly, it was found that particular DNA atomic sites are frequently the targets of drug covalent actions. In actuality, DNA can be considered a macromolecular receptor for these medications. The various classes of DNA-acting anticancer medications are listed [9]. Some intercalate (daunorubicin and doxorubicin, for example) or groove-bind to DNA to form noncovalent complexes with DNA (such as Distamycin A). Covalent bonds are created between DNA and substances like cisplatin, Mitomycin C, and ecteinascidins. Last but not least, some antibiotics can cause DNA backbone cleavages (for instance, duocarmycin, bleomycin/phleomycin, and enediyne) [8,10,11]. Due to their excellent pharmacological activity, particularly their anticancer properties, heterocyclic compounds (particularly those containing nitrogen atoms) have dominated organic chemistry for more than a century [12]. In medicinal chemistry, the sulfonamide moiety has gained prominence as an effective bioisostere of the carboxylic group [13].

**Figure 1:**
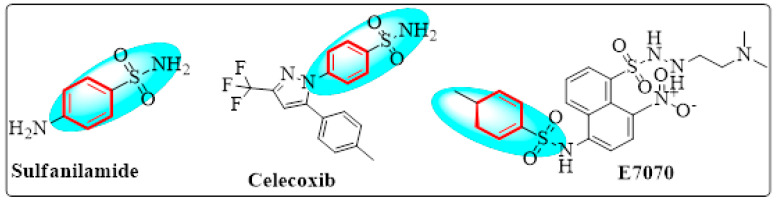
Previously reported sulphonamide derivatives as anti-cancer agents [17,18,19].

A hydrogen bond network comparable to that produced by the carboxylic group may be generated by the sulfonamide motif. Additionally, the distance between oxygen atoms in both of these functional groups is nearly identical [14,15]. As the carboxylic group’s bioisostere, it may avoid some of the carboxylic group’s drawbacks, such as limited passive diffusion across biological membranes, toxicity, and metabolic instability [16]. A broad range of sulfonamide derivatives (Figure 1) have been developed, with biological actions ranging from anti-bacterial to anti-fungal, antioxidant, anti-inflammatory, antidiabetic, and anti-cancer [17,18]. As a consequence, the sulfonamide moiety has found widespread acceptance in medicinal chemistry. These compounds can inhibit cancer cell development, induce apoptosis, and disrupt the cell cycle [19].

In this study, a combination of in-vitro and in-silico methodologies was used to evaluate the anti-cancer potential of two sulphonamide compounds, with a focus on their interactions with three critical cancer proteins: p53, caspase, and NF-κB. This research also draws attention to p53, caspases, and NF-κB as important players in the progression of cancer and prospective therapeutic targets. p53 targeting is a promising strategy for the development of anti-cancer drugs since mutations in this gene are linked to many different types of cancer [20,21,22]. Caspases are also involved in apoptosis, and their activation is a critical step in inducing programmed cell death [23]. NF-κB dysregulation has been linked to cancer development, and targeting NF-κB has emerged as a possible technique for developing anti-cancer drugs [24,25].

Possible modes of action and anticancer potential of N-ethyl toluene-4-sulphonamide (**8a**) and 2,5-Dichlorothiophene-3-sulphonamide (**8b**) were assessed by experimental and computational methodologies. The studied compounds’ cytotoxicity was evaluated by in-vitro cell line experiments. This involves exposing cancer cells to the drug candidate and measuring its effect on cell growth and viability. Computational methods and in-vitro experiments were used to examine the compounds’ properties and their predicted interactions with proteins involved in cancer. Molecular docking was used to estimate the binding modes and affinities of drug candidates with target proteins (p53, caspase, and NF-κB). The electrical structure and properties were fine-tuned with the use of density functional theory (DFT). Time-dependent changes in protein-ligand interactions were studied using molecular dynamic (MD) simulations to learn more about the stability of protein-ligand complexes. Thus, combined, in-vitro and computational investigations improved our understanding of the potential efficacy and mechanism of action of possible anticancer lead compounds.

## 2. Result and Discussions

### 2.1. Biological Evaluation

#### 2.1.1. In-Vitro Cytotoxic Activity

Both compounds (**8a**) and (**8b**) exhibited significant cytotoxic activity against all cancer cell lines. The IC_50_ values ranged between 1.62 and 12.74 µM (Table 1). Compound (**8b**) showed the most potent cytotoxic activity against HeLa, MCF-7, and MDA-MB-231 with IC_50_ values (4.62 to 7.21 µM). These results are comparable to reference compounds (Cisplatin and doxorubicin). Compound (**8a**) also showed significant cytotoxic activity and the IC_50_ values were between (10.91 to 19.22 µM). These results indicate that both compounds have promising anticancer activity and may serve as potential anticancer drugs.

Compound (**8a**) showed the highest IC_50_ values against HeLa at 10.9 ± 1.01 μM. It also exhibited IC_50_ of 19.22 ± 1.67 and 12.21 ± 0.93 μM against MDA-MB231 and MCF-7, respectively. Whereas IC_50_ values for Compound (**8b**) showed the greatest promise in inhibiting MDA-MB231 and MCF-7 cell proliferation when compared to Compound (**8a**). Interestingly, the Compound (**8b**) exhibited comparable results with reference drugs. It can be hypothesized that the compound having a thiophene ring exhibited maximum anticancer potential and appeared as a better compound than the compound containing a phenyl ring attached to the sulphonamide group. DNA investigations corroborated the findings and provided further evidence that these chemicals could serve as a starting point for the production of further potential anticancer drugs.

#### 2.1.2. UV-Visible Spectroscopy-Based DNA Binding Studies

**Figure 2:**
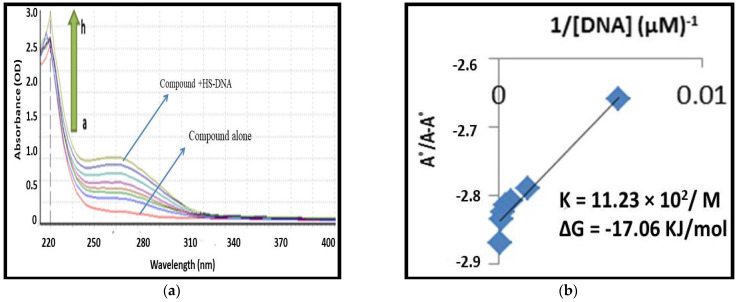
(**a**) Depiction of UV-visible spectral responses of N-Ethyltoluene-4-sulfonamide (**8a**) in the presence and absence of various concentrations of the previously stated HS-DNA. The arrowhead denotes the rising DNA concentration. (**b**) Ao-A/Ao vs. 1/[DNA] graph for calculating binding constants.

**Figure 3:**
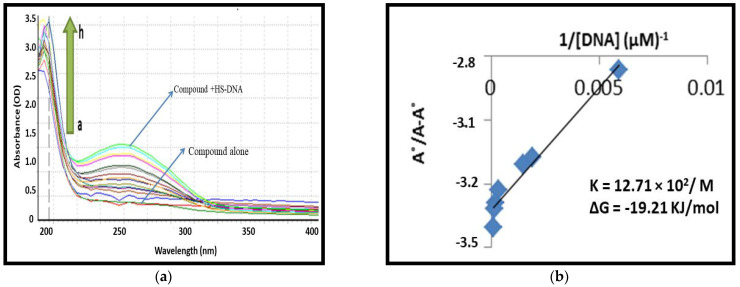
(**a**) Depiction of UV-visible spectral responses of 2,5-Dichlorothiophene-3-sulfonamide (**8b**) in the presence and absence of various concentrations of the previously stated HS-DNA. The arrowhead denotes the rising DNA concentration. (**b**) Ao-A/Ao vs. 1/[DNA] graph for calculating binding constants.

The mechanistic behavior of (**8a**) and (**8b**) were further explored by performing their interaction studies with mammalian DNA (HS-DNA) [26,27,28]. The spectra of both compounds were obtained with a fixed concentration of 10 µM of (**8a**) or (**8b**) in the absence and presence of a different concentration of HS-DNA (0–240 µM). Both (**8a**) and (**8b**) displayed enhanced absorbance with no shift in band locations, while the HS-DNA concentration was increased (Figure 2 and Figure 3). Hence, the hyperchromic effect expressed the non-covalent interaction of HS-DNA with both compounds [28,29].

Interestingly, (**8b**), which was found best during the MTT assay, also showed maximum DNA interactions with Gibbs free energy ΔG° −19.51 KJ/mol. In addition, compound (**8a**) also showed promising results but fewer binding interactions. Hence, it supports the cell viability results.

#### 2.1.3. Density Functional Theory Calculations

The geometrical structure (**8a**) and (**8b**) calculations were done at the B3LYP/6-31G* level of theory. In addition, their structural geometries were tuned for a maximum energy gradient with minimum occurrences of imaginary frequency, and the electronic properties were evaluated. The optimal and reactive parameters for both compounds are listed in Table 2.

**Figure 4:**
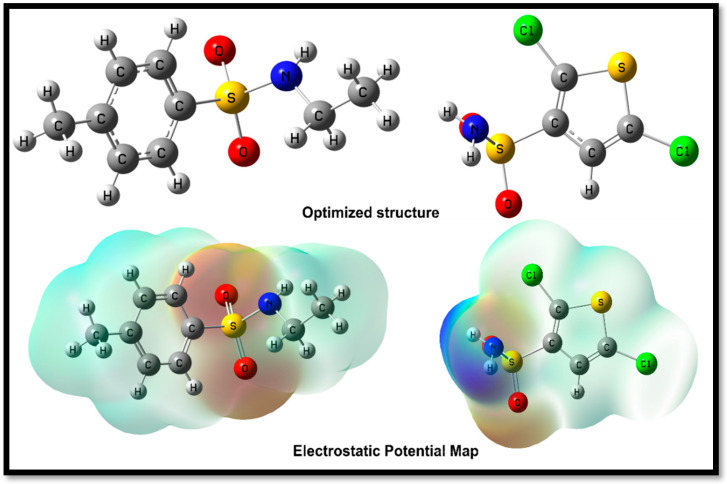
The optimized structures of (**8a**) (**left**) and (**8b**) (**right**) alongside electrostatic potential map.

In this study, two compounds, N-ethyl toluene-4-sulphonamide (**8a**) and 2,5-Dichlorothiophene-3-sulfonamide (**8b**)**,** were analyzed for their inhibitory potential. The optimization energy, polarizability, dipole moment, potential ionization energy, affinity energy, electron-donating power, electron-accepting power, and electrophilicity were determined for both compounds. Compound (**8a**) was found to have an optimization energy of −953.809912 Hartree, a polarizability of 300.221 a.u, a dipole moment of 6.201887 Debye, and a potential ionization energy of 0.258 eV. It was determined that (**8a**) had an electron receiving the power of 0.246 and an electron-donating power of 0.199. In addition, 2,5-Dichlorothiophene-3-sulfonamide (**8b**) was found to have a higher optimization energy of −2075.743816 Hartree, a lower polarizability of 110.149294 a.u, a dipole moment of 3.618891 Debye, and a potential ionization energy of 0.2711 eV. The electron-donating power of 2,5-Dichlorothiophene-3-sulfonamide (**8b**) was found to be 0.261, while its electron-accepting power was 0.346. The electrophilicity of both compounds was calculated as the difference between the electron-donating and electron-accepting power, with N-Ethyltoluene-4-sulfonamide (**8a**) having a Δω ± value of 0.047, while Compound 2,5-Dichlorothiophene-3-sulfonamide (**8b**) has a Δω ± value of 0.085. The electrostatic potential map of the compound highlights regions of high and low electrostatic potential. The red areas have high electron-attracting potential because of the electronegative oxygen atoms present. The blue areas have a lesser electron-donating potential, possibly because of the presence of hydrogen atoms. These maps provide crucial insight into a molecule’s electron reactivity and distribution (Figure 4).

The compounds, (**8a**) and (**8b**), were analyzed to determine their electronic properties. The results suggest that **8a** had a HOMO energy of −0.258 eV and a LUMO energy of −0.046 eV with a band gap of 0.211 eV. On the other hand, 2,5-Dichlorothiophene-3-sulfonamide (**8b**) had a HOMO energy of −0.271 eV and a LUMO energy of −0.080 eV, yielding a band gap of 0.191 eV. The energy needed to excite an electron from the HOMO to the LUMO level is measured by the band gap of a molecule, which is a significant parameter in determining its electrical conductivity.

The chemical hardness (η) was measured as 0.106 eV and 0.096 eV for (**8a**) and (**8b**), respectively. The lower the value of chemical hardness, the higher the reactivity will be. The chemical potential (μ) (a measure of the ability of a molecule to donate electrons) for (**8a**) was −0.152 eV, while that of (**8b**) was −0.176 eV. The electrophilicity index (ω) of N-ethyl toluene-4-sulphonamide (**8a**) was 0.110 eV, while that of 2,5-Dichlorothiophene-3-sulfonamide (**8b**) was 0.161 eV. The electrophilicity index quantifies a molecule’s propensity to receive electrons, with a greater number indicating a stronger tendency to do so. The chemical softness (S) of (**8a**) was 4.736 eV and that of (**8b**) was 5.233 eV. Chemical softness is a measure of the ability of a molecule to undergo chemical reactions, with higher values indicating a higher reactivity. The electronegativity (X) of N-ethyl toluene-4-sulphonamide (**8a**) was 0.152 eV, and that of 2,5-Dichlorothiophene-3-sulfonamide (**8b**) was 0.176 eV. One of the most important factors in a molecule’s reactivity is its electronegativity, or its capacity to attract electrons. These features provide insight into possible applications of these compounds (Table 3).

**Figure 5:**
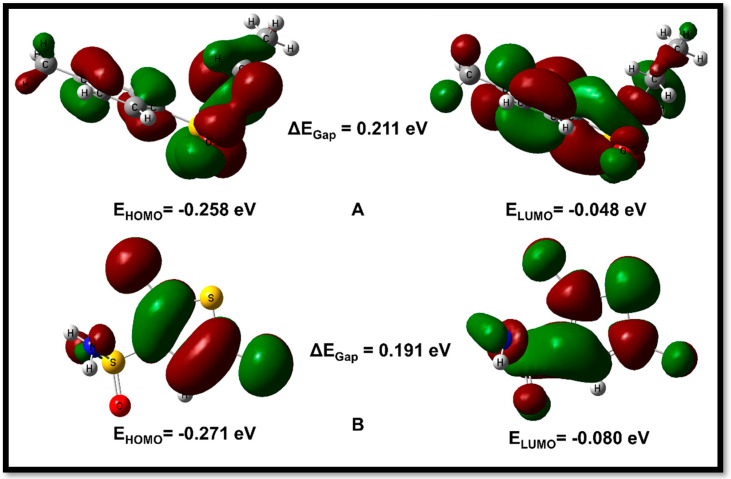
HOMO/LUMO orbitals of compound N-ethyl toluene-4-sulphonamide (**8a**) (**A**) and compound 2,5-Dichlorothiophene-3-sulfonamide (**8b**) (**B**).

The orbital study of (**8a**) reveals that the sulphonamide group is the primary contributor of HOMO, with help from the hydrophobic benzene ring and the methyl group. The LUMO is primarily localized on the toluene ring, which gives the compound an electrophilic character. In compound (**8b**), the HOMO orbitals are primarily located on the dichloro-substituted thiophene ring, while the LUMO orbitals are mainly located on the sulphonamide fragment of the molecule, with some slight delocalization also observed on the thiophene ring. These findings provide information about the electrical structure and chemical reactivity of these molecules (Figure 5).

#### 2.1.4. Molecular Docking Studies

Molecular docking is a computational method widely used in drug discovery and development to predict the binding mode and binding affinity of a small molecule (ligand) to a target protein or nucleic acid (receptor). The aim of molecular docking is to identify potential drug candidates that can bind specifically to a target protein and modulate its activity. In this study, Compounds (**8a**) and (**8b**) were tested for their ability to intercalate with DNA. Caspase-3 plays a critical role in cellular apoptosis. The inflammation, cell survival, and cell proliferation are regulated by NF-B, a transcription factor. Therefore, the field of cancer research must assess how these drugs interact with these cancer proteins. It was observed that (**8b**) formed considerable interactions with all studied proteins and intercalated DNA forcefully. Notably, (**8b**) interacted with caspase-3, and hydrophilic and hydrophobic interactions occurred between them with a best docking score of −5.9 kcal/mol, whereas (**8a**) inhibited p53 protein greatly with a docking score of −5.7 kcal/mol. In addition, the molecular docking analysis was performed for three different complexes, namely p53-cisplatin, NF-κB-cisplatin, and Caspase-3-cisplatin, as well as the DNA-Doxorubicin and DNA-cisplatin complexes. The docking scores for each complex were determined, indicating the binding affinity between the ligand and the respective protein or nucleic acid.

In the case of p53-cisplatin and DNA-Doxorubicin complexes, moderate-to-strong binding affinities were observed, with several hydrogen bonding and hydrophobic interactions identified between the ligand and the respective nucleotide or amino acid residues. These interactions are critical for stabilizing the complex and enhancing the binding affinity. In contrast, weak binding affinities were observed for the NF-κB-cisplatin, Caspase 3-cisplatin, and DNA-cisplatin complexes, with fewer hydrogen bonding and hydrophobic interactions identified. These results suggest that these complexes may not be ideal drug targets; further optimization of the ligands or target proteins may be required to improve binding affinity. These results provide valuable information about anticancer potential and mode of action for (**8a**) and (**8b**) (Table 4).

##### Interpretation of Molecular Interactions

Molecular docking analysis was used to assess the nature of the interaction between (**8a**) and Caspase-3. The binding energy of compound (**8a**) to Caspase-3 was calculated to be −4.9 kcal/mol. The interaction involved one hydrogen bond with the asparagine residue Asn208 (3.5 Å bond length). Additionally, hydrophobic interactions were observed with multiple residues, including Asp253, Trp206, Arg207, Trp214, Asn208, Phe247, Glu246, Glu248, Phe250, Phe252, and Ser251. These interactions could be crucial in ensuring the stability of (**8a**) binding to Caspase-3.

**Figure 6:**
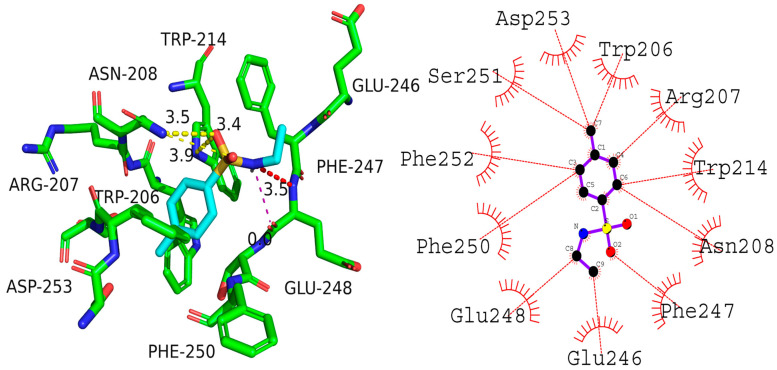
Presumed 2D and 3D binding mode of compound N-ethyl toluene-4-sulphonamide (**8a**) with Caspase-3.

The hydrogen bonding and hydrophobic interactions between (**8a**) and Caspase-3 indicated that the compound could be a lead molecule for the development of new Caspase-3 inhibitors. Caspase-3 is an essential protein involved in the apoptosis process and has an important role in carcinogenesis. Compound (**8a**) showed promising cancer cell growth inhibition after creating a complex with the Caspase-3 enzyme. Once the complex is established with Caspase-3, the protein’s function may be inhibited (Figure 6).

Docking of (**8b**) and Caspase-3 revealed a docking score of −5.9 kcal/mol, which is higher than the score obtained for (**8a**) (−5.9 kcal/mol). Several amino acid residues in the protein were found to be involved in the interactions when (**8b**) was analyzed for its interactions with the protein. Gly165 formed a hydrogen bond of 2.92 Å bond length with it, whereas His121, Arg207, Thr255, Tyr204, Thr166, and Gly122 were involved in the hydrophobic interactions.

**Figure 7:**
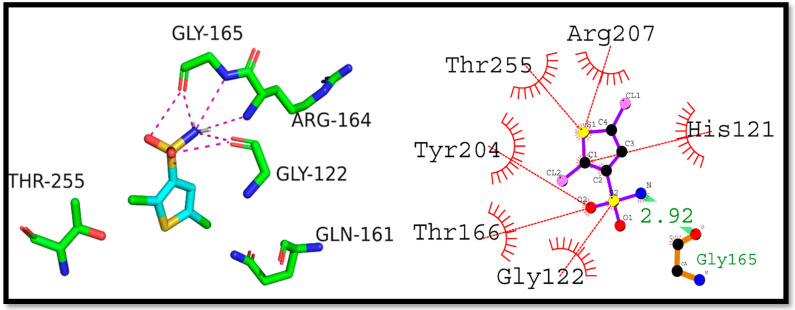
2,5-Dichlorothiophene-3-sulfonamide (**8b**) hypothesized to interact with Caspase-3 in 2D and 3D space.

In comparing the bonds between (**8a**) and (**8b**), Compound (**8a**) interacted with Asn208, while (**8b**) interacted with Gly165. Further, both compounds interacted with Arg207 and Thr166 with hydrophobic interactions. The data suggest that both compounds may engage in protein-ligand interactions involving hydrogen bonding and hydrophobic bonds. However, the interactions between the two compounds are distinct, including the particular residues involved. These findings could provide insights into the design of more potent inhibitors of Caspase-3 (Figure 7).

**Figure 8:**
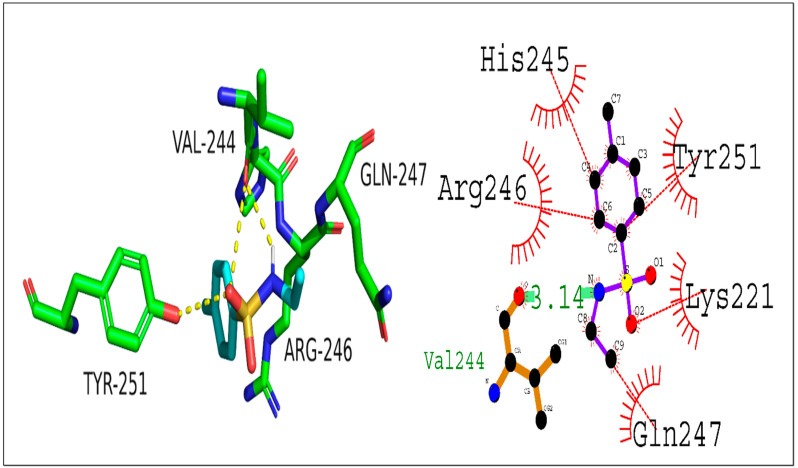
N-ethyl toluene-4-sulphonamide (**8a**) hypothesized to interact with NF-κB in 2D and 3D space.

Further, Compound (**8a**) interaction with the NF-κB transcription factor was studied using molecular docking. The docking score was found to be −5.2 kcal/mol and a hydrogen bond (3.14 Å) was observed between Val244 and (**8a**). In addition, hydrophobic interactions were also observed with His245, Arg246, Gln247, Lys221, and Tyr251. These interactions could increase the complex’s stability, suggesting that Compound (**8a**) could be used as a starting point for designing NF-κB inhibitors (Figure 8).

**Figure 9:**
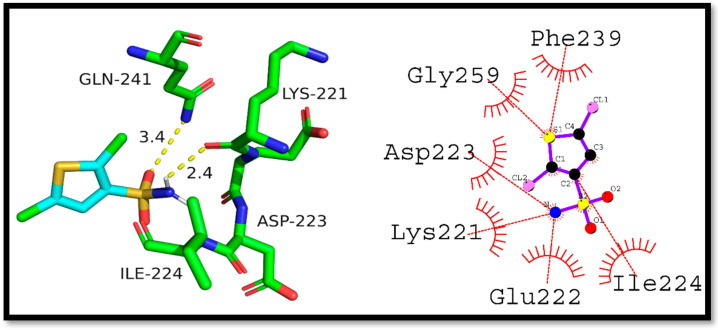
Presenting 2D and 3D interaction of 2,5-Dichlorothiophene-3-sulfonamide (**8b**) against NF-κB.

The interaction of (**8b**) with NF-κB was studied, and the interaction had a docking score of −5.8 kcal/mol. Two hydrogen bonds were observed in the interaction between Gln241 and Lys221, with respective bond lengths of 3.4 and 2.4 Å. The hydrophobic interactions were observed with Phe239, Gly259, Asp223, Lys221, Glu222, and Ile224 residues. The results infer that the compound (**8b**) may have the potential as an NF-κB inhibitor (Figure 9).

**Figure 10:**
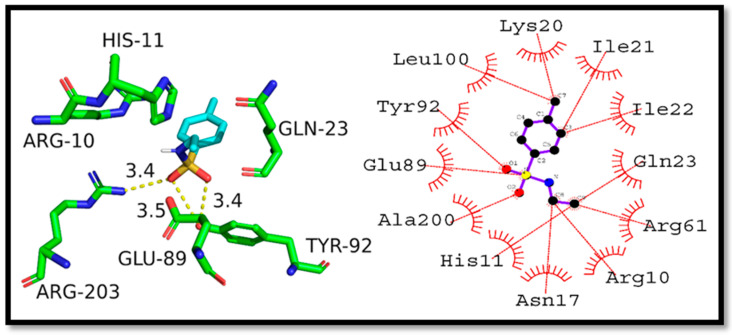
Illustrating the binding orientation of compound N-ethyl toluene-4-sulphonamide (**8a**) against p53.

The molecular docking study of Compound (**8a**) with p53 showed a good binding affinity and −5.7 kcal/mol as the docking score. The compound formed three hydrogen bonds with Arg203, Tyr92, and Glu89 residues of the p53 protein with a hydrogen bond length of 3.4, 3.4, and 3.5 Å, respectively. The hydrophobic interactions were observed between the compound and Lys20, Leu100, Tyr92, Ala200, His11, Asn17, Arg10, Arg61, Gln23, Ile22, and Ile21 residues of the protein. Because these residues contribute significantly to the formation of hydrogen bonds and hydrophobic interactions with Compound **8b**, there is a possibility that the compound could impair the activity of p53. As a result, it has the potential to serve as a promising lead molecule in the research and development of new p53 inhibitors (Figure 10).

**Figure 11:**
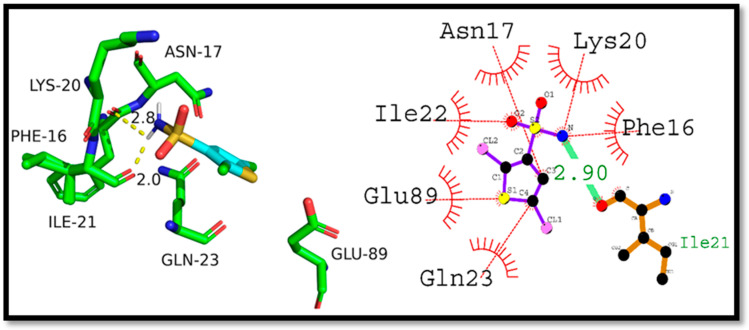
Illustration of binding orientation of Compound 2,5-Dichlorothiophene-3-sulfonamide (**8b**) against p53.

In contrast to Compound (**8a**), Compound (**8b**) showed a relatively lower docking score of −5.1 kcal/mol with the p53 protein. The analysis of the interaction pattern indicated a hydrogen bond between the carbonyl group of (**8b**) and the Ile21 residue of the p53 protein with a 2.90 Å bond length. Moreover, Phe16, Lys20, Asn17, Ile22, Glu89, and Gln23 had hydrophobic interactions with (**8b**). Hence, it was concluded that (**8b**) has a weaker binding affinity with p53 compared to (**8a**) and was attributed to the absence of key hydrogen bonding residues and the shorter hydrogen bond length in (**8b**). Therefore, improving (**8b**) binding affinity with p53 protein will necessitate further refinement of its chemical structure (Figure 11).

**Figure 12:**
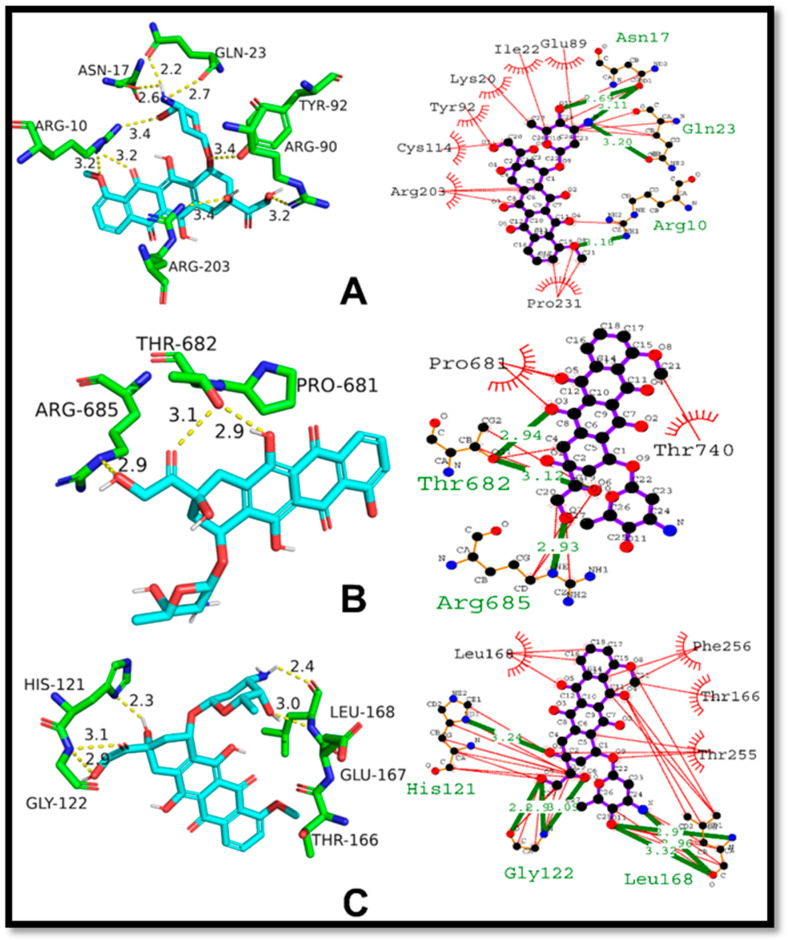
Molecular docking studies of standard Doxorubicin against respective proteins; (**A**) 2D and 3D binding mode of Doxorubicin against p53, (**B**) 2D and 3D binding mode of Doxorubicin against NF-κB, (**C**) 2D and 3D binding mode of Doxorubicin against Caspase-3.

The presented Figure 12 below displays the results of molecular docking analysis for various complexes, namely p53-Doxirubicin, NF-κB-Doxorubicin, and Caspase 3-Doxorubicin. The docking score represents the strength of the binding affinity between the ligand (Doxorubicin) and the respective protein complex.

In the p53-Doxirubicin complex, the docking score of −10.53 kcal/mol indicates a robust interaction between the two molecules. The ligand established hydrogen bonding with the Asn17, Gln23, and Arg10 residues, with a bond length of 2.69, 3.20, and 3.18 angstroms, respectively. Furthermore, several hydrophobic interactions were noticed with Glu89, Ile22, Lys20, Tyr92, Cys114, Arg203, and Pro231 residues. These interactions are crucial for stabilizing the protein-ligand complex and enhancing its binding affinity. For the NF-κB-Doxorubicin complex, the docking score of −4.52 kcal/mol suggests a moderate interaction between the two molecules. The ligand formed hydrogen bonds with Thr682 and Arg685 residues, with a bond length of 2.94 and 2.93 angstroms, respectively. In addition, several hydrophobic interactions were detected with Pro681 and Thr740 residues. In the case of the Caspase 3-Doxorubicin complex, the docking score of −9.49 kcal/mol represents a strong interaction between the two molecules. The ligand created hydrogen bonds with His121, Gly122, and Leu168 residues, with a bond length of 3.24, 2.2, and 2.96 angstroms, respectively. Several hydrophobic interactions were also observed with Leu168, Phe256, Thr166, and Thr255 residues. Hydrogen bonding interactions play a significant role in stabilizing the protein-ligand complex. These bonds form when a hydrogen atom of the ligand interacts with an electronegative atom of the protein residue, such as nitrogen or oxygen. Such interactions are essential in maintaining the ligand’s orientation and conformation within the protein’s active site. Hydrophobic interactions also play a crucial role in protein-ligand binding, as they contribute to the overall stability of the complex. These interactions occur between nonpolar regions of the ligand and protein residues, such as aliphatic chains and aromatic rings. Hydrophobic interactions also contribute to the specificity of ligand binding, as specific protein residues may have unique hydrophobic pockets that only interact with specific ligands.

**Figure 13:**
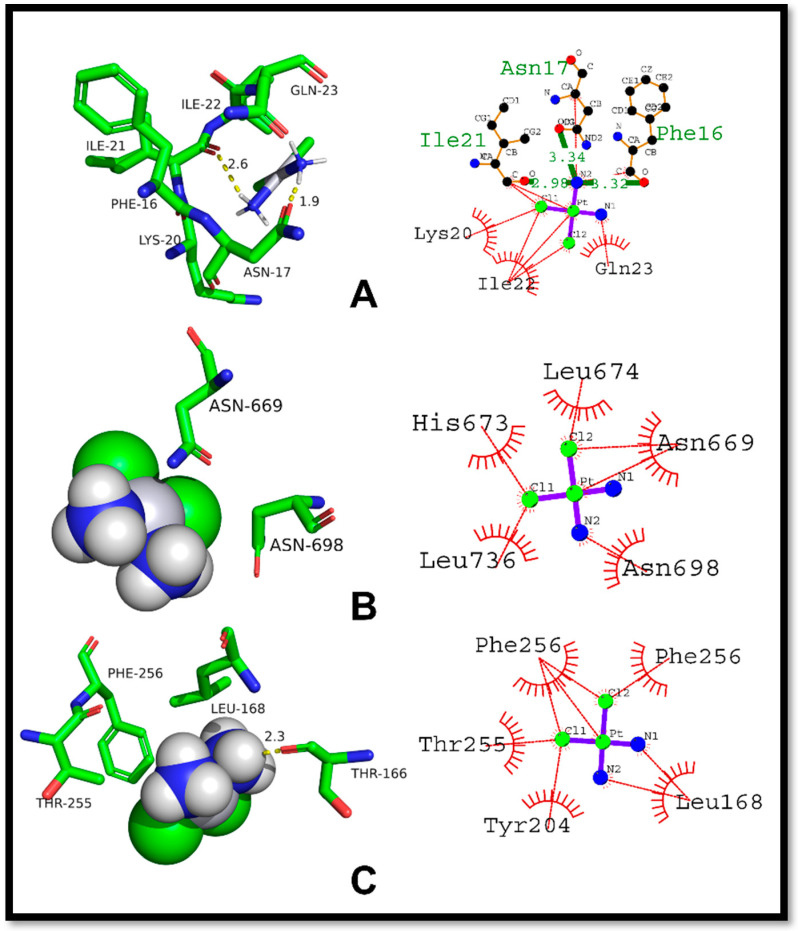
Molecular docking studies of standard Cisplatin against respective proteins; (**A**) 2D and 3D binding mode of Cisplatin against p53, (**B**) 2D and 3D binding mode of Cisplatin against NF-κB, (**C**) 2D and 3D binding mode of Cisplatin against Caspase-3.

The presented Figure 13 below showcases the results of molecular docking analysis for three distinct protein-ligand complexes: p53-cisplatin, NF-κB-cisplatin, and Caspase 3-cisplatin. The docking score reflects the strength of the binding affinity between the ligand, cisplatin, and its respective protein complex. Regarding the p53-cisplatin complex, the docking score was −3.27 kcal/mol, indicating a moderate interaction between the two molecules. The ligand showed hydrogen bonding with Asn17, Ile21, and Phe16 residues, with a bond length of 3.34, 2.98, and 3.32 angstroms, respectively. Additionally, several hydrophobic interactions occurred with Leu20, Ile22, and Gln23 residues. These interactions contribute to the stabilization of the protein-ligand complex, thus strengthening the binding affinity. As for the NF-κB-cisplatin complex, the docking score was −2.38 kcal/mol, indicating a weak interaction between the two molecules. No hydrogen bonding residues were observed for this complex. Nevertheless, several hydrophobic interactions took place with Leu674, Ile673, Leu736, Asn698, and Asn669 residues. In the case of the Caspase 3-cisplatin complex, the docking score was −2.57 kcal/mol, also indicating a weak interaction between the two molecules. No hydrogen bonding residues were found for this complex either. However, several hydrophobic interactions occurred with Phe256, Thr255, Tyr204, Leu168, and Phe256 residues.

##### DNA Molecule Intercalation

**Figure 14:**
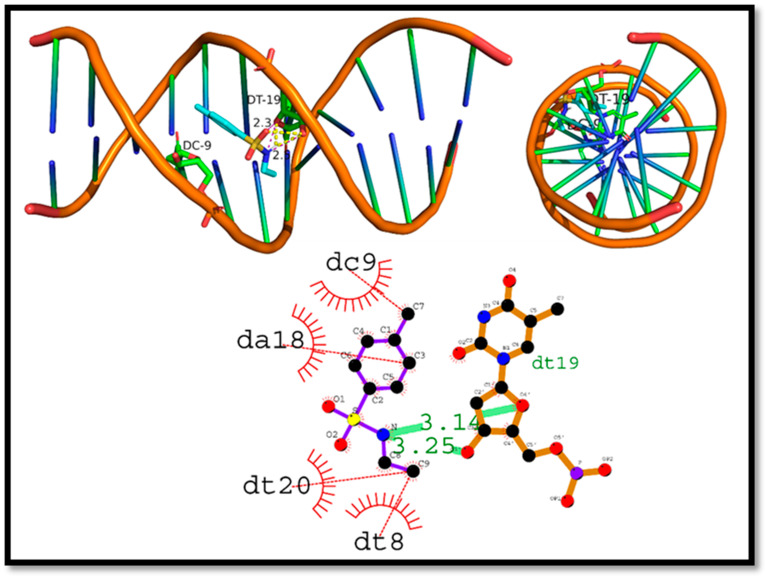
Intercalation of DNA groove by compound N-ethyl toluene-4-sulphonamide (**8a**).

The interaction of (**8a**) with DNA, had −5.1 kcal/mol as the docking score. The compound formed hydrogen bonds with the nucleotide bases, with the deoxythymidine residues (Dt19 and Dt19) forming hydrogen bonds with lengths of 3.14 and 3.25 angstroms, respectively. The hydrophobic interactions were found with deoxycytidine (Dc9), deoxyadenosine (Da18), deoxythymidine (Dt20), and deoxythymidine (Dt8). These results indicate that Compound N-ethyl toluene-4-sulphonamide (**8a**) can interact with the DNA molecule, potentially affecting its structure and function. In addition, Compound N-ethyl toluene-4-sulphonamide (**8a**) was completely buried inside the DNA helix, which demonstrates a strong intercalation of DNA. Such interactions could have implications for the use of (**8a**) in the treatment of diseases involving DNA, such as cancer. To fully comprehend, additional research is required to establish the mechanism of action of (**8a**) to DNA (Figure 14).

**Figure 15:**
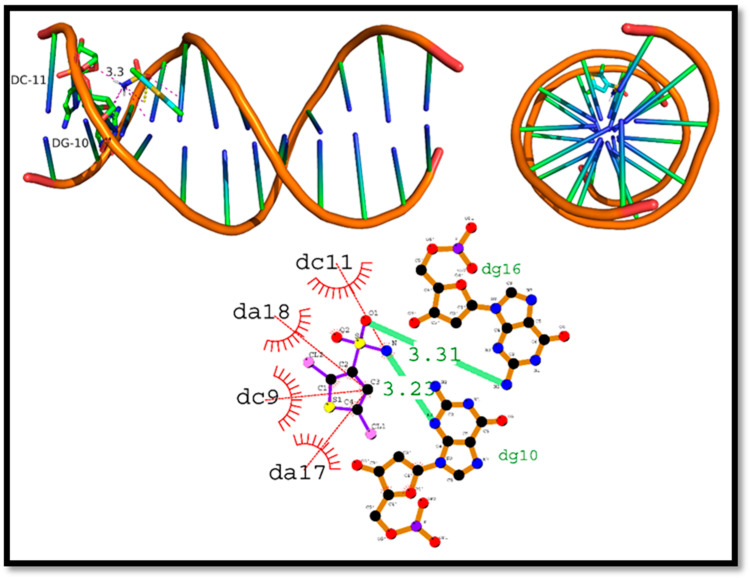
Intercalation of DNA groove by Compound 2,5-Dichlorothiophene-3-sulfonamide (**8b**).

The interaction of (**8b**) with DNA, had a docking score of −5.6 kcal/mol, and the two major hydrogen bonds were identified as Dg10 and Dg16, with bond lengths of 3.23 and 3.31 2.90 Å, respectively. In addition, four hydrophobic interaction residues were identified, including Dc11, Da18, Dc9, and Dc17. The results support the idea that (**8b**) can interact with DNA via both hydrogen and hydrophobic interaction (Figure 15).

The DNA-Doxorubicin complex analysis revealed that the ligand (Doxorubicin) established hydrogen bonds with five DNA nucleotide residues. These nucleotides included two Thymine (Dt20) residues, one Thymine (Dt19) residue, one Deoxyadenosine (Da8) residue, and one Deoxyguanosine (Dg7) residue, with bond lengths ranging from 2.66 to 3.26 angstroms. The formation of these hydrogen bonds is critical for stabilizing the complex and promoting a stronger binding affinity between the two molecules. In addition, the analysis also detected several hydrophobic interactions between Doxorubicin and DNA nucleotide residues, such as Deoxyadenosine (Da6), Deoxycytosine (Dc21), Deoxyadenosine (Da5), Deoxyguanosine (Dg4), and Deoxyguanosine (Dg22). These interactions played a crucial role in increasing the overall stability of the complex, leading to a higher binding affinity between the two molecules.

**Figure 16:**
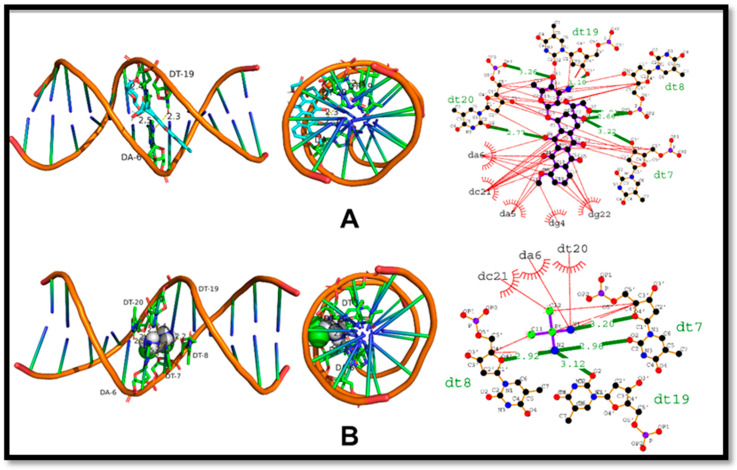
The intercalation of DNA by standard molecules; (**A**) intercalation of DNA by Doxorubicin, (**B**) Intercalation of DNA by Cisplatin.

Figure 16 illustrates the results of the molecular docking analysis of the DNA-cisplatin complex. The computed docking score for this complex was −3.79 kcal/mol, suggesting a moderate interaction between the two molecules. Cisplatin ligand formed hydrogen bonds with three DNA nucleotide residues, including two Thymine (Dt8 and Dt7) residues and one Thymine (Dt19) residue, with bond lengths of 2.92, 2.96, and 3.12 angstroms, respectively. These hydrogen bonding interactions are vital in stabilizing the DNA-cisplatin complex and strengthening the binding affinity between the two molecules. Furthermore, the analysis revealed several hydrophobic interactions between cisplatin and DNA nucleotide residues, including Thymine (Dt20), Deoxyadenosine (Da6), and Deoxycytosine (Dc21) residues. These hydrophobic interactions also played a critical role in stabilizing the complex and significantly enhancing the binding affinity between cisplatin and DNA.

#### 2.1.5. Molecular Dynamics Simulation

##### Root Mean Square Deviation Plot

The best conformations of (**8a**) and (**8b**) were investigated using molecular dynamic simulation to determine stability against their respective proteins. The compound (**8a**) demonstrated the highest binding affinity against p53, whereas (**8b**) was producing a strong inhibition of Caspase-3. The MDS helped to analyze the interactions at the molecular level and the residues identified that play a major role in the complex formation.

**Figure 17:**
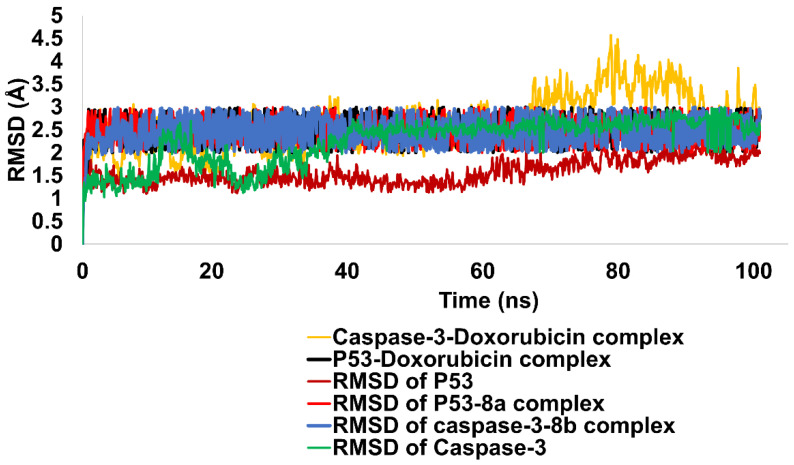
Illustration of RMSD pattern for Apo proteins (Caspase-3 and p53) and all four complexes along with standard Doxorubicin.

Using the RMSD studies the stability of apoprotein and Caspase-3-**8b** complexes were evaluated. Throughout the MD simulations investigations, the apoprotein (Caspase-3) displayed a significant stability pattern ranging from 1.5 to 2.5 Å. Slight rearrangements were observed initially at around 20 ns. However, they were stabilized during the early run. The Caspase-3-**8b** combination remained stable throughout the predicted trajectory. The RMSD of ligand-proteins was 2.6 angstroms on average. The strength of the interaction between the ligand and protein determined whether they could form a stable complex. The importance of hydrogen bonds and hydrophobic interactions to the complex’s stability was observed. The hydrogen bonds were responsible for the structural stability of the Caspase-3-**8b** complex, suggesting their importance for stable complex formations. The stability of the complex was enhanced by the hydrophobic interactions and aided in maintaining the complex’s stability. The progression of the relative mean square deviation (RMSD) for Caspase-3 and Caspase-3-**8b** complexes is depicted in Figure 17.

Similarly, the stability of the apoprotein (p53) and the p53-(**8a**) complex was analyzed using RMSD. The RMSD values for the apoprotein ranged anywhere from 1.2 to 2.2 Å. About halfway through the simulation, the trajectory stabilized and re-equilibrated. However, the p53-(**8a**) complex showed some slight rearrangements as the simulated trajectory progressed. In the first 10 ns, the RMSD of the p53-(**8a**) complex was 1.8 Å, then it increased to a maximum of 2.8 Å before leveling off and reaching equilibrium. Variations in the RMSD values are indicative of conformational changes in the p53-(**8a**) complex that occurred during the simulation. While there were fluctuations, they were within a reasonable range, so the complex’s overall stability was maintained (Figure 17).

The standard Doxorubicin was contrasted with the simulated complexes of Compounds (**8a**) and (**8b**). It was interesting to note that the complexes of Caspase-3-Doxorubicin and p53-Doxorubicin displayed a considerable stability pattern that was similar to the substance under examination in the current study. Caspase 3-Doxorubicin RSMD was 2.8 angstroms, whereas the average RMSD of the p53-Doxorubicin complex was 2.5 angstroms. Whereas Caspase-3-doxorubicin showed only minor oscillations before becoming stable and equilibrated, p53-doxorubicin complex stood out for its considerable stability pattern. Figure 17 shows the evolution of RMSD across all complexes.

**Figure 18:**
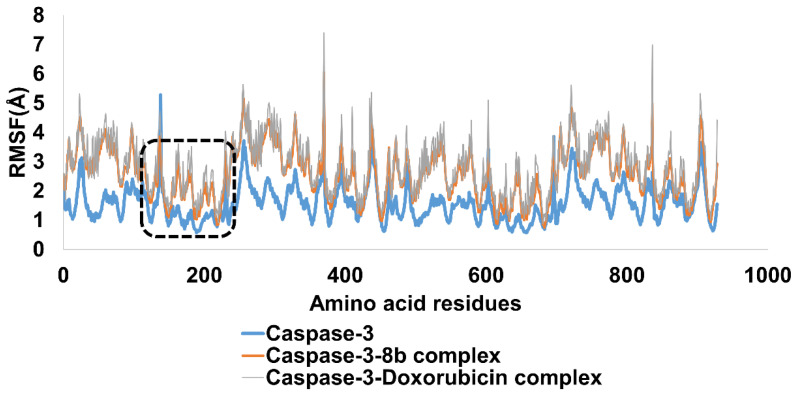
RMSF pattern for c alpha atoms of Caspase-3, Caspase-3-(**8b**) complex, and Caspase 3-Doxorubicin complex.

The complex remained stable during the simulation, as determined by the Root Mean Square Fluctuation (RMSF). Amino acid deviation from its typical position can be inferred from the RMSF values. The outcomes confirmed that the protein maintained its structural integrity throughout the simulation. The protein-ligand complex was found to be thermodynamically stable, demonstrating its strength. The RMSF analysis offers information about the stability of the protein-ligand interaction, and the studied complex demonstrated that the important amino acid residues interact with the ligand. The RMSF value for Caspase-3 was found to be at an average of 2.4 Å and is considered acceptable. In addition, RMSF analysis of Caspase-3 in complex with Compound (**8b**) and doxorubicin is also presented in Figure 18. It was notable that residues were a fluctuation of Caspase-3 in a complex with Compound (**8b**) was comparable to standard Doxorubicin. The average RMSF for Caspase-3-(**8b**) and Caspase-3-Doxorubicin was 2.3 and 2.4 angstroms, respectively. The amino acid residues range from 100–200 was in in contact with both compounds i.e., (**8b**) and doxorubicin. It was encouraging that these residues exhibited fewer fluctuations and remained stable. The results from this study can help in the development of new drugs targeting specific proteins in biological systems (Figure 18).

**Figure 19:**
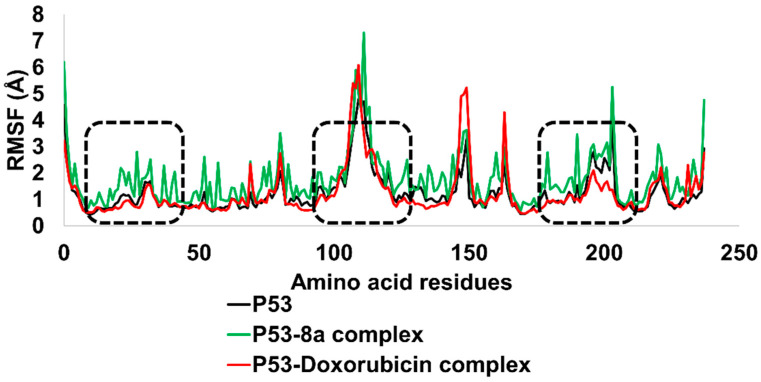
RMSF analysis of p53 in contact with N-ethyl toluene-4-sulphonamide (**8a**) and Doxorubicin.

The RMSF for the p53-(**8a**) complex exhibited stability for the majority part of the simulated trajectory. The RMSF values as high as 4.7 were observed for residues 100–130. The amino acids in contact with ligand (**8a**) were stable throughout the simulation, even though this peak appeared. Thus, the ligand can maintain its interactions with the amino acid residues in the binding site of the p53-(**8a**) complex, suggesting that the complex is relatively stable. In addition, RMSF values of p53 in complex with Doxorubicin are also presented in Figure 19. The amino acid residues range from 20–25, 100–140, and 160–200; these were in contact with both ligands. The RMSF study corroborated the findings of the DFT calculations and MD simulations, which showed that the p53-N-ethyl toluene-4-sulphonamide (**8a**) complex is chemically reactive and stable (Figure 19).

**Figure 20:**
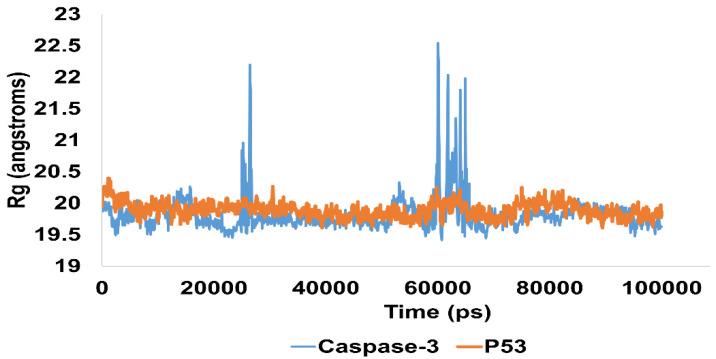
Radius of gyration.

The compactness of a protein or polymer can be measured using the radius of gyration (Rg), which is calculated as the root-mean-square distance of its particles from the center of mass. In this study, the Rg of the protein Caspase-3 and p53 were determined and found to fluctuate between 19.25, 19.75, and 20–20.3 angstroms, respectively. These fluctuations in Rg can be indicative of changes in the protein’s compactness resulting from alterations in its conformation or interactions with its surroundings. For instance, when exposed to denaturing agents like high temperatures or chemical denaturants, the protein may unfold, leading to an increase in Rg. Conversely, when stabilized by favorable interactions like ligand binding or protein-protein interactions, the protein may become more compact, leading to a decrease in Rg. Figure 20 illustrates the radius of gyration.

##### MMGBSA Free-Binding Energy Analysis

The MMGBSA (Molecular Mechanics Generalized Born Surface Area) is a popular technique for predicting the binding free energy of protein-ligand complexes. This method determines the binding free energy by computing the difference between the free energies of the protein-ligand complex and the unbound components (i.e., the protein and the ligand). In MMGBSA energy analysis, the free-binding energies of protein, ligand, and the protein-ligand complex are estimated by the following equation [30]:

*G* = *E_bnd_* + *E_el_* + *E_vdW_* + *G_pol_* + *G_np_* − *TS*

where *E_bnd_* refers to bond energy, *E_el_* refers to electrostatic energy, and *E_vdW_* represents van der Waals interactions. In the current study, Poisson-Boltzmann calculations were performed using the internal GBSA solver in mmpbsa_py_energy. All units are represented in kcal/mol. An MM-GBSA energy analysis is illustrated in Table 5.

In this study, the Schrodinger Python script was used to calculate the binding free energies of four protein-ligand complexes, namely p53-cisplatin, p53-doxorubicin, p53-(**8a**), and caspase3-(**8b**). The results of the study show that the p53-(**8a**) complex has the lowest CFBE value of −5508.8 kcal/mol, while the Caspase-3-**8b** complex has a slightly lower CFBE value of −5822.1 kcal/mol. This implies that the interactions between the protein and ligand molecules in these two complexes are relatively weak. On the other hand, the CFBE values for the p53-Doxorubicin and p53-Cisplatin complexes are −1022.6 kcal/mol and −1002 kcal/mol, respectively, indicating that these complexes have stronger interactions. The data also provide information on the different energy components that contribute to the overall CFBE, including Columb, hydrogen bond, and covalent energies. Among the four complexes, p53-(**8a**) has the highest Columb energy (−8235.2 kcal/mol), indicating strong electrostatic interactions between the protein and ligand molecules. The highest hydrogen bond energy is observed in the p53-Doxorubicin complex (−420.2 kcal/mol), which suggests that the protein-ligand complex has strong hydrogen-bonding interactions. The covalent energy is the lowest for the p53-Cisplatin complex (−232.9 kcal/mol), indicating a relatively weak covalent interaction. In summary, the data provide valuable insights into the energetics of protein-ligand complexes, which can be used to understand the binding mechanism and guide the development of more effective ligands. However, it is important to exercise caution when interpreting the results as they are subject to various assumptions and approximations used in the calculations. The detailed values are provided in Table 5.

**Figure 21:**
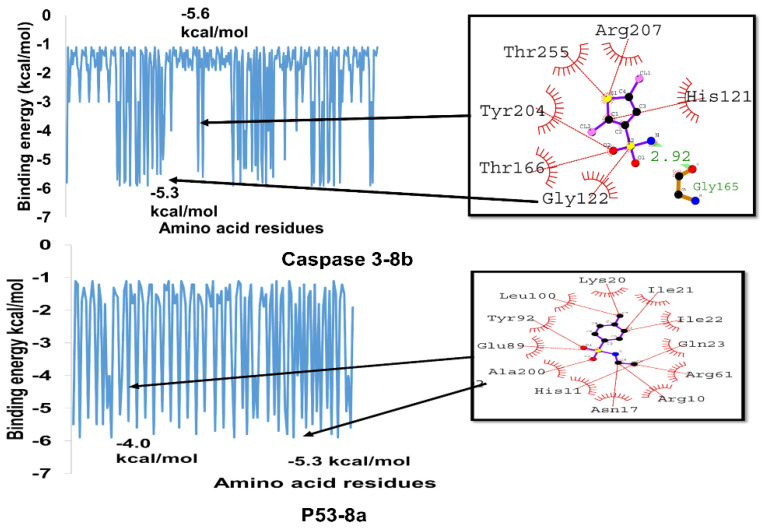
Amino acid residues wise binding energies of Caspase-3-**8b** and p53-(**8a**) complex.

The binding energies of each amino acid residue of Caspase-3 and p53 are calculated; they range from −1.0 to −5.9 kcal/mol, as illustrated in Figure 21. It can be seen from the readings’ oscillations between these two extremes that some residues have a higher affinity for binding than others. The variation in the residues’ binding energies serves as a visual cue to the intricate nature of protein-ligand interactions. The shape, size, and chemical characteristics of the ligand and the protein surface, as well as the amino acid makeup of the protein’s binding site, all have an impact on binding energy.

**Figure 22:**
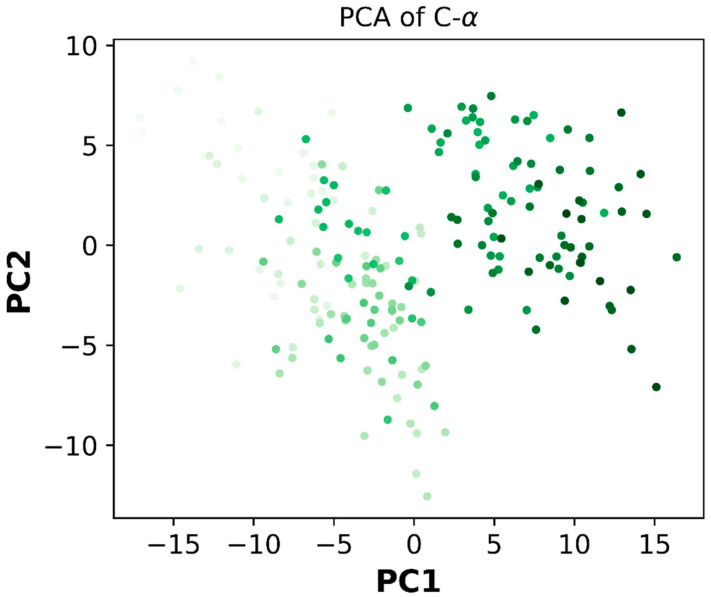
Principle component analysis for Caspase -3 (dark green colored dots indicate PCA at 100 ns).

PCA (Principal Component Analysis) is a statistical method for reducing the number of dimensions in a dataset while preserving the majority of the data. This is accomplished by locating the principal components, which are linear combinations of the initial variables that account for the majority of the variance in the data. PCA can be used to examine the structure and operation of the protein Caspase-3, which is a protease enzyme involved in apoptosis or programmed cell death (Figure 22).

## 3. Materials and Methods

### 3.1. Assay for Cell Viability

The two compounds, N-ethyl toluene-4-sulphonamide (**8a**) and 2,5-Dichlorothiophene-3-sulfonamide (**8b**)**,** were explored for their potential to fight cancer against three different cell lines using cell viability assay: MDA-MB231, MCF-7 (human breast cancer cell lines), and HeLa (a human cervical cancer cell line). To perform the assay, 10 × 10^4^ cells were added to each well of a plate, which was followed by 10 μL of (**8a**) or (**8b**). The plates were then incubated at 37 °C and 5% CO_2_ for 24 h. After incubating the plates at 37 °C for 4 h, 10 μL of MTT reagent were added. The reaction was then stopped by adding 10% sodium dodecyl sulfate. Background absorbance was calculated at 625 nm and optical density was determined at 575 nm using FLUOstar Omega microplate reader (BMG Labtech, Ortenberg, Germany) [31,32]. The IC_50_ and percent growth inhibition of (**8a**) or (**8b**) were determined using GraphPad Prism Software version 5.0. Doxorubicin and cisplatin were used as positive controls in the cell viability assay. Potential anticancer agent effectiveness was assessed by comparing the findings of (**8a**) and (**8b**) to the positive controls [32].

### 3.2. Spectrophotometric DNA Binding Analysis

The binding interaction of N-ethyl toluene-4-sulphonamide (**8a**) and 2,5-Dichlorothiophene-3-sulfonamide (**8b**) with DNA using UV-visible spectrophotometry was evaluated. To prepare the solutions for the experiment, 10% DMSO solutions of the compounds were prepared. The stock solution for Lyophilized Herring sperm DNA was prepared by weighing and then dissolving it in distilled water. The DNA concentration was calculated using the 260/280 absorbance ratio. With a ratio between 1.6 and 1.9, it was determined that the DNA was of sufficient purity for analysis. The investigational compounds were added to the range of HS-DNA concentrations (40 μM, 80 μM, 120 μM, 160 μM, 200 μM, and 240 μM). After the incubation (30 min in a dark place), UV absorption spectra were recorded using a FLUOstar Omega microplate reader [33,34]. Changes in the (**8a**) or (**8b**) spectra in the presence of DNA suggested a binding interaction between the chemicals and DNA. The binding constants and modes of interaction between the compounds and DNA were measured from the obtained spectra.

### 3.3. Computational Investigations

#### 3.3.1. Density Functional Theory Calculations

DFT calculations are widely used to study the electronic properties and structural stability of molecules. In this study, the DFT calculations were used to evaluate the electronic properties of N-ethyl toluene-4-sulphonamide (**8a**) and 2,5-Dichlorothiophene-3-sulfonamide (**8b**) using the Gaussian 09W program [35,36]. The B3LYP functional correlation and the 6-31G* basis set were used to optimize the geometries and evaluate the stability of the compounds [37].

The electron density of the compounds was analyzed using local and global reactivity descriptors, as well as the frontier molecular orbitals [38]. The chemical sensitivity, ionization potential, and reactivity of the compounds were evaluated based on their electron densities. The findings indicated that the compounds had a high ion-attracting capacity and were promising ligand choices [39,40]. The GaussView 06 [41] analysis provided suitability as potential ligands through the assessment of their properties. In general, the DFT calculations elucidated useful information on the electrical characteristics and structural stability of the compounds, which might be used in the creation of effective anticancer medicines.

#### 3.3.2. Molecular Docking Studies

Molecular docking was used to evaluate the non-covalent interactions of compounds N-ethyl toluene-4-sulphonamide (**8a**) and 2,5-Dichlorothiophene-3-sulfonamide (**8b**) as inhibitors against various cancer proteins, including Caspase-3, NF-κB, p53, and DNA. The study used proteins sourced from the Protein Data Bank (PDB: PDB IDs: 3DEI [42], 1NFI [43], 3DCY [44,45], and 127D [46] respectively, www.rcsb.com accessed on 15 February 2023) and prepared them for docking by removing water and hetero-atoms, adding polar hydrogen atoms, and incorporating Gasteiger charges [30,47]. The grid box of one angstrom spacing was set for molecular docking purposes. In addition, the attributes of co-crystal ligand were used for production run. The coordinates for the NF-κB protein were obtained from the Protein Data Bank (PDB), and the x, y, and z attributes were set to −5.373126, 65.514536, and 45.078298, respectively. The dimensions of the p53 protein were set to 17.000000, 41.837500, and −3.621000, while the dimensions for the DNA were set to 9.927250, 23.023156, 8.770906 and −46.798037, 15.020000, and −21.901333 for Caspase 3. To learn how the ligand would attach to the enzyme’s active site, the proteins’ protonation states were also altered. Electrostatic interactions between the ligand and the protein are influenced by the protonation status of amino acids within the active site, which influences binding affinity and specificity. To obtain a precise depiction of the binding interactions, molecular docking, and MDS need to consider the protonation states of the protein and ligand in their analyses.

The compounds (**8a**), (**8b**), and standard doxorubicin and cisplatin were prepared for direct docking in AutoDock Vina [48]. The grid box was enlarged to include the targeted proteins’ active sites. The exhaustiveness was set to Default 8, and the number of modes was set to 100 to obtain precise docking findings. The co-crystal ligand was docked to verify the docking procedure. If the RMSD between the original and rebuilt position was fewer than two angstroms, the docking method was considered successful [47].

#### 3.3.3. Molecular Dynamics Simulations

MDS studies on the protein-ligand complex were performed on Desmond. Each complex was simulated in the TIP3P solvent model and was used for 50 ns. [49]. Restoration of the system to its original state was performed with counter sodium chloride (NaCl) ions 0.15 M. The OPLS3 forcefield was utilized during the simulation [50]. The atomic motion was integrated by this forcefield while it was being exposed to periodic boundary conditions. To prevent any collisions of atoms from occurring, an initial strategy of 2000 steps for minimizing energy was used. The system was brought to equilibrium in an isothermal and isobaric environment at 300 K and 1.01 bar (NPT) [51,52]. To focus on interactions at shorter ranges, van der Waals calculations were performed using a cutoff distance of 10 angstroms. The Nose-Hoover thermostat and the Martyna-Tobias-Klein barostat were employed to maintain pressure and temperature during the simulation [53]. When integrating the motion equations, a 2-fs time step was employed. The production run lasted 100 ns, and the simulated trajectories were saved at 50 ps intervals. Particle Mesh Ewald was used to investigate electrostatic interactions with high accuracy and reliability [54,55]. The Desmond simulation interaction diagram protocol was used to analyze the simulated trajectories of the protein-ligand complexes. The MM-GBSA and principle component analysis were performed as discussed earlier [30].

## 4. Conclusions

The free-binding energy is affected by both electrostatic and non-electrostatic forces, and Compound (**8b**) was determined to have the greatest potential as an anticancer agent. Molecular docking studies suggest that Compound (**8b**) has the strongest affinity for binding Caspase-3, while (**8a**) has the strongest affinity for p53. DFT calculations that supported the findings demonstrated that both compounds have good chemical reactivity with the localization of the HOMO and LUMO orbitals at specific regions of the molecules, supporting the claim that 2,5-Dichlorothiophene-3-sulfonamide (**8b**) is a potential anti-cancer compound. Docking and molecular dynamic (MD) simulations added further support to the stability of both compounds in their binding sites. Both compounds exhibited better docking scores than cisplatin and comparable docking scores to Doxorubicin. These results highlight the importance of integrating computational and experimental methods when developing novel therapies for the treatment of a wide range of diseases and also provide valuable information about the potential of these molecules as Caspase-3 and p53 inhibitors.

## Figures and Tables

**Table 1:** Cytotoxic activity of (**8a**) and (**8b**) against human HeLa, MDA-MB-231, and MCF-7 cancer cell lines.

Compound	IC_50_ (µM) ± SEM		
	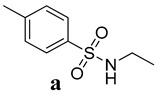		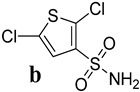
	HeLa	MDA-MB231	MCF-7
(**8a**)	10.9 ± 1.01	19.22 ± 1.67	12.21 ± 0.93
(**8b**)	7.2 ± 1.12	4.62 ± 0.13	7.13 ± 0.13
Doxorubicin	4.21 ± 0.22	6.82 ± 0.59	7.32 ± 0.81
Cisplatin	2.64 ± 0.13	2.27 ± 0.24	4.63 ± 0.21

**Table 2:** Optimization parameters in DFT for compounds of (**8a**) and (**8b**).

Compound	Optimization Energy (Hartree)	Polarizability a.u (α)	Dipole Moment (Debye)	Potential Ionization I (eV)	Affinity A(eV)	Electron Donating Power (ω−)	Electron Accepting Power (ω+)	Electro Philicity (Δω±)
(**8a**)	−953.809912	300.221	6.201887	0.258	0.04684	0.047	0.199	0.246
(**8b**)	−2075.743816	110.149294	3.618891	0.2711	0.0800	0.085	0.261	0.346

**Table 3:** Global and local reactivity descriptors of N-ethyl toluene-4-sulphonamide (**8a**) and N-ethyl toluene-4-sulphonamide (**8a**).

Compound	E_HOMO_ (eV)	E_LUMO_ (eV)	∆E_gap_ (eV)	Chemical Hardness (η)	Chemical Potential (μ)	Electrophilicity Index (ω)	Chemical Softness (S)	Electronegativity (X)
(**8a**)	−0.258	−0.046	0.211	0.106	−0.152	0.110	4.736	0.152
(**8b**)	−0.271	−0.080	0.191	0.096	−0.176	0.161	5.233	0.176

**Table 4:** The molecular interactions observed during molecular docking.

Complex	Docking Score (kcal/mol)	Hydrogen Bonding Residues	Hydrogen Bond Length (Angstroms)	Hydrophobic Interactions Residues
Caspase-3-N-ethyl toluene-4-sulphonamide (**8a**)	−4.9	Asn208	3.5	Asp253, Trp206, Arg207, Trp214, Asn208, Phe247, Glu246, Glu248, Phe250, Phe252, Ser251
Caspase-3-2,5-Dichlorothiophene-3-sulfonamide (**8b**)	−5.9	Gly165	2.92	His121, Arg207, Thr255, Tyr204. Thr166, Gly122
NF-κB-N-ethyl toluene-4-sulphonamide (**8a**)	−5.2	Val244	3.14	His245, Arg246, Gln247, Lys221, Tyr251
NF-κB-2,5-Dichlorothiophene-3-sulfonamide (**8b**)	−5.8	Gln241, Lys221	3.4, 2.4	Phe239, Gly259, Asp223, Lys221, Glu222, Ile224
p53-N-ethyl toluene-4-sulphonamide (**8a**)	−5.7	Arg203, Tyr92, Glu89	3.4, 3.4, 3.5	Lys20, Leu100, Tyr92, Ala200, His11, Asn17, Arg10, Arg61, Gln23, Ile22, Ile21
p53-2,5-Dichlorothiophene-3-sulfonamide (**8b**)	−5.1	Ile21	2.90	Phe16, Lys20, Asn17, Ile22, Glu89, Gln23
DNA-N-ethyl toluene-4-sulphonamide (**8a**)	−5.1	Dt19, Dt19	3.14, 3.25	Dc9, Da18, Dt20, Dt8
DNA-2,5-Dichlorothiophene-3-sulfonamide (**8b**)	−5.6	Dg10, Dg16	3.23, 3.31	Dc11, Da18, Dc9, Dc17
p53-Doxirubicin	−10.53	Asn17, Gln23, Arg10	2.69, 3.20, 3.18	Glu89, Ile22, Lys20, Tyr92, Cys114, Arg203, Pro231
NF-κB-Doxorubicin	−4.52	Thr682, Arg685	2.94, 2.93	Pro681, Thr740
Caspase 3-Doxorubicin	−9.49	His121, Gly122, Leu168	3.24, 2.2, 2.96	Leu168, Phe256, Thr166, Thr255
DNA-Doxorubicin	−9.93	Dt20, Dt20, Dt19, Dt8, Dt7	2.77, 3.26, 3.10, 2.66, 3.22	Da6, Dc21, Da5, Dg4, Dg22
p53-cisplatin	−3.27	Asn17, Ile21, Phe16	3.34, 2.98, 3.32	Ly20, Ile22, Gln23
NF-κB-cisplatin	−2.38	-	-	Leu674, Jis673, Leu736, Asn698, Asn669
Caspase 3-cisplatin	−2.57	-	-	Phe256, Thr255, Tyr204, Leu168, Phe256
DNA-cisplatin	−3.79	Dt8, Dt19, Dt7	2.92, 3.12, 2.96	Dt20, Da6, Dc21

**Table 5:** The MMGBSA data for selected complexes.

Complexes	Complex Free Binding Energy (kcal/mol)	Columb Energy (kcal/mol)	Hydrogen Bond Energy (kcal/mol)	Covalent Energy (kcal/mol)
p53-(**8a**)	−5508.8	−8235.2	−123.1	−6012
Caspase-3-(**8b**)	−5822.1	−8421.4	−165.5	−7233
p53-Doxorubicin	−1022.6	−1523.9	−420.2	−1232
p53-Cisplatin	−1002	−503.5	−54.2	−232.9

## Data Availability

Data will be available on request to corresponding author.
